# Adaptive Target Birth Intensity Multi-Bernoulli Filter with Noise-Based Threshold

**DOI:** 10.3390/s19051120

**Published:** 2019-03-05

**Authors:** Xiaolong Hu, Hongbing Ji, Long Liu

**Affiliations:** School of Electronic Engineering, Xidian University, Xi’an 710071, China; huxiaolong0@126.com (X.H.); longliu@xidian.edu.cn (L.L.)

**Keywords:** measurement likelihood, multi-Bernoulli, multi-target tracking, random finite sets, target birth model, threshold

## Abstract

Adaptively modeling the target birth intensity while maintaining the filtering efficiency is a challenging issue in multi-target tracking (MTT). Generally, the target birth probability is predefined as a constant and only the target birth density is considered in existing adaptive birth models, resulting in deteriorated target tracking accuracy, especially in the target appearing cases. In addition, the existing adaptive birth models also give rise to a decline in operation efficiency on account of the extra birth modeling calculations. To properly adapt the real variation of the number of newborn targets and improve the multi-target tracking performance, a novel fast sequential Monte Carlo (SMC) adaptive target birth intensity cardinality balanced multi-target multi-Bernoulli (CBMeMBer) filter is proposed in this paper. Through adaptively conducting the target birth probability in a pre-processing step, which incorporates the information of current measurements to correct the pre-setting of the target birth probability, the proposed filter can truly adapt target birth cases and achieve better tracking accuracy. Moreover, the implementation efficiency can be improved significantly by employing a measurement noise-based threshold in the likelihood calculations of the multi-target updating. Simulation results verify the effectiveness of the proposed filter.

## 1. Introduction

The core objective in multi-target tracking (MTT) is to estimate the states of multiple moving targets, based on obtained data with spontaneous appearance and disappearance of different targets. Through generalizing the single-target recursive filter to the multi-target tracking situation, the random finite set (RFS)-based Bayesian filter [1] was proven to be appropriate for multi-target tracking. To reduce the algorithm complexity of the RFS Bayes multi-target filter, a series of solutions such as the probability hypothesis density (PHD) filter [2,3], the cardinalized PHD (CPHD) filter [4,5], the cardinality balanced multi-target multi-Bernoulli (CBMeMBer) filter [6,7], and the generalized labeled multi-Bernoulli (GLMB) filter [8,9,10] were developed. The PHD filter propagates the first moment of the multi-target density, and the CPHD filter is a generalization of the PHD filter that jointly propagates the moment and cardinality distribution to provide better tracking accuracy. The CBMeMBer filter propagates the multi-Bernoulli distribution to approximate the multi-target density, and the GLMB filter is a generalization of the CBMeMBer filter that propagates a generalized labeled multi-Bernoulli distribution to jointly provide better performance and target trajectories. Compared to the PHD and CPHD filters which are synonymous, the multi-Bernoulli representations have advantages in cases that ask for individual target existence probabilities or sequential Monte Carlo (SMC) implementations. In the SMC multi-Bernoulli filters, the clustering algorithm to extract the states of targets can be avoided, while it is required in the SMC PHD and CPHD filters.

The RFS-based filters were widely applied in various tracking scenarios. In these scenarios, environment descriptions, e.g., the newborn target [11,12,13,14,15], the target motion models [16,17], the unknown target [18,19], the unknown process noise statistics [20,21], the unknown measurement noise statistics [22,23], the target measurement models [24,25], and the detection and clutter rate uncertainty [26,27], are of great importance. It is worth noting that the description of newborn targets attracted intensive attention over the recent years, which is typically called the target birth modeling. “Birth” means that a target appears in the observation region. In the RFS-based filters, the target birth model is expressed as an intensity component [1] and applied at each filtering step. The target birth intensity consists two parts: the target birth density and the target birth probability, which represent the positions and the corresponding possibilities that newborn targets may appear, respectively. Modeling the target birth intensity is to capture the targets that enter the field of view (FOV) in real time. However, the indeterminacy of target birth makes the problem intractable.

So far, most researches on the RFS-based Bayesian filters required a priori location information of newborn targets and took the target birth intensity as fixed values. However, in practice, such as in the area of air target tracking, if the positions of airports are unavailable or the targets emerge from other positions, serious errors may occur. Meanwhile, the rough assumption fixing the number of newborn targets as a constant is improper and may cause severe degradation of the estimation accuracy. In the existing literature, adaptive target birth modeling is rarely discussed. In Reference [11], an adaptive target birth model was applied in the PHD and CPHD filters by grouping the targets into newborn and surviving sets, whose target birth density was generated through the measurements of the previous time step. Originated from Reference [11], the adaptive birth model was applied in the CBMeMBer and GLMB filters [12,13], respectively, using a simplified structure. In Reference [14], an evaluating step of multiple measurements and states including newborn targets was proposed. In Reference [15], a newborn track detection and state estimation method was proposed using the Bernoulli RFS based on the sequential change in measurements. However, with an aim to remove the requirement of the prior location information about newborn targets, the existing adaptive birth modeling methods focus on the target birth density, while still setting the target birth probability as a constant. On the other hand, compared with the traditional fixed birth model, the time consumption of the filters applying adaptive birth models increases dramatically, since these birth models are more complex and need to be integrated into the filtering recursion at every time step.

In this paper, we propose an improved multi-target Bayesian filter based on the CBMeMBer framework which can adapt unknown target birth cases including both density and probability. The target birth density is modeled by the measurements received at the previous moment, which is similar to the method in Reference [13], while the target birth probability is calculated by a pre-processing step using the current received measurements. This CBMeMBer filter with the new adaptive target birth intensity not only eliminates the requirement of prior birth information, but also avoids the coarse assumption of the number of newborn targets. Thus, the proposed filter can achieve a better estimation accuracy and can be used in more tracking scenarios. To reduce the heavy computational burden incurred by the adaptive target birth intensity, a threshold-based method is introduced, which can eliminate most of the redundant measurement-updating calculations. Different from the widely used thresholds for single-target tracking [28,29] or requiring target–measurement association [29,30,31], the proposed threshold is determined by the measurement noise and operates on the distance between the state of particles and measurements, without any requirements for track continuity. The improved adaptive target birth intensity CBMeMBer filter with the threshold method is shown to be able to achieve better tracking performance with reduced computational burden.

The remainder of this paper is organized as follows: the fundamentals of the standard CBMeMBer filter are reviewed in Section 2. The adaption of the CBMeMBer filter to accommodate the unknown target birth intensity, and its fast filtering recursion with a novel threshold are elaborated on in Section 3 and Section 4, respectively. Simulation results are presented in Section 5, and conclusions are given in Section 6.

## 2. Cardinality Balanced Multi-Target Multi-Bernoulli Filter

This section reviews the standard CBMeMBer filter, which is a full Bayesian filter within the multi-Bernoulli framework and was used in many fields such as sensor networks [32], audio data [33], visual tracking [34], etc.

We assume that, at time k, the RFSs [1] of the multi-target state $X_{k}$ and the multi-target observation $Z_{k}$ in the state and observation space [1] χ and Z can be written as

(1) $$X_{k}=\left\{x_{k,1},\ldots ,x_{k,N(k)}\right\}\subset \chi ,$$

(2) $$Z_{k}=\left\{z_{k,1},\ldots ,z_{k,M(k)}\right\}\subset Z.$$

The recursions of the multi-target posterior density are based on the framework of the multi-target Bayesian filter [1], where the RFSs are used to express the set-valued random variables.

In the CBMeMBer filters, Bernoulli and multi-Bernoulli RFSs are basic elements. A Bernoulli RFS [6] X means a dyadic distribution. With probability r and density p, X can be written as

(3) $$\pi \left(X\right)=\{\begin{array}{cc} 1-r & X=\emptyset \\ r\cdot p\left(x\right) & X=\left\{x\right\} \end{array}.$$

A multi-Bernoulli RFS $X=\cup_{i=1}^{M}X^{\left(i\right)}$ represents an integration of independent Bernoulli RFSs $X^{\left(i\right)}$, which can be written as ${\left\{\left(r^{\left(i\right)},p^{\left(i\right)}\right)\right\}}_{i=1}^{M}$. The sum of the existence probability $r^{\left(i\right)}$ describes the cardinality of the multi-Bernoulli RFS. A detailed introduction can be found in References [1,6].

The CBMeMBer filter [6] is derived based on the following assumptions:

1. The evolution of each target and the generation of each observation are all independent;
2. The clutter is independent of the observations of targets and follows a Poisson distribution;
3. One target can generate at most one observation at each scan;
4. Target birth is multi-Bernoulli and is independent of target survival.

The propagation of the CBMeMBer filter is presented below.

Suppose that the posterior multi-target density at time k−1 follows a multi-Bernoulli distribution given by

(4) $$\pi_{k-1}={\left\{\left(r_{k-1}^{\left(i\right)},p_{k-1}^{\left(i\right)}\right)\right\}}_{i=1}^{M_{k-1}}.$$

Then, the predicted multi-target density is derived by combining the multi-Bernoulli distributions representing the newborn targets and the surviving targets, which can be written as

(5) $$\pi_{k|k-1}={\left\{\left(r_{\Gamma ,k}^{\left(i\right)},p_{\Gamma ,k}^{\left(i\right)}\right)\right\}}_{i=1}^{M_{\Gamma ,k}}\cup {\left\{\left(r_{P,k|k-1}^{\left(i\right)},p_{P,k|k-1}^{\left(i\right)}\right)\right\}}_{i=1}^{M_{k-1}},$$

where ${\left\{\left(r_{\Gamma ,k}^{\left(i\right)},p_{\Gamma ,k}^{\left(i\right)}\right)\right\}}_{i=1}^{M_{\Gamma ,k}}$ is the prediction of newborn targets at time k, and ${\left\{\left(r_{P,k|k-1}^{\left(i\right)},p_{P,k|k-1}^{\left(i\right)}\right)\right\}}_{i=1}^{M_{k-1}}$ is the prediction of surviving targets at time k, with

(6) $$r_{P,k|k-1}^{\left(i\right)}=r_{k-1}^{\left(i\right)}\langle p_{k-1}^{\left(i\right)},p_{S,k}\rangle ,$$

(7) $$p_{P,k|k-1}^{\left(i\right)}\left(x\right)=\frac{\langle f_{k|k-1}\left(x|\cdot \right),p_{k-1}^{\left(i\right)}p_{S,k}\rangle }{\langle p_{k-1}^{\left(i\right)},p_{S,k}\rangle },$$

where $p_{S,k}\left(\eta \right)$ and $f_{k|k-1}\left(\cdot |\eta \right)$ are the survival probability and the Markov transition density. The total number of predicted Bernoulli distributions is given by $M_{k|k-1}=M_{k-1}+M_{\Gamma ,k}$. Therefore, the predicted multi-target density at time k is still a multi-Bernoulli distribution with

(8) $$\pi_{k|k-1}={\left\{\left(r_{k|k-1}^{\left(i\right)},p_{k|k-1}^{\left(i\right)}\right)\right\}}_{i=1}^{M_{k|k-1}}.$$

After the prediction obtained, the approximated posterior multi-target density can be written as

(9) $$\pi_{k}\approx {\left\{\left(r_{L,k}^{\left(i\right)},p_{L,k}^{\left(i\right)}\right)\right\}}_{i=1}^{M_{k|k-1}}\cup {\left\{\left(r_{U,k}\left(z\right),p_{U,k}\left(\cdot ;z\right)\right)\right\}}_{z\in Z_{k}},$$

where the legacy tracks are expressed by

(10) $$r_{L,k}^{\left(i\right)}=r_{k|k-1}^{\left(i\right)}\frac{1-\langle p_{k|k-1}^{\left(i\right)},p_{D,k}\rangle }{1-r_{k|k-1}^{\left(i\right)}\langle p_{k|k-1}^{\left(i\right)},p_{D,k}\rangle },$$

(11) $$p_{L,k}^{\left(i\right)}\left(x\right)=p_{k|k-1}^{\left(i\right)}\left(x\right)\frac{1-p_{D,k}\left(x\right)}{1-\langle p_{k|k-1}^{\left(i\right)},p_{D,k}\rangle },$$

with detection probability $p_{D,k}\left(x\right)$. The measurement update tracks are expressed by

(12) $$r_{U,k}\left(z\right)=\frac{\sum_{i=1}^{M_{k|k-1}}\frac{r_{k|k-1}^{\left(i\right)}\left(1-r_{k|k-1}^{\left(i\right)}\right)\langle p_{k|k-1}^{\left(i\right)},\psi_{k,z}\rangle }{{\left(1-r_{k|k-1}^{\left(i\right)}\langle p_{k|k-1}^{\left(i\right)},p_{D,k}\rangle \right)}^{2}}}{\kappa_{k}\left(z\right)+\sum_{i=1}^{M_{k|k-1}}\frac{r_{k|k-1}^{\left(i\right)}\langle p_{k|k-1}^{\left(i\right)},\psi_{k,z}\rangle }{1-r_{k|k-1}^{\left(i\right)}\langle p_{k|k-1}^{\left(i\right)},p_{D,k}\rangle }},$$

(13) $$p_{U,k}\left(x;z\right)=\frac{\sum_{i=1}^{M_{k|k-1}}\frac{r_{k|k-1}^{\left(i\right)}}{1-r_{k|k-1}^{\left(i\right)}}p_{k|k-1}^{\left(i\right)}\left(x\right)\psi_{k,z}\left(x\right)}{\sum_{i=1}^{M_{k|k-1}}\frac{r_{k|k-1}^{\left(i\right)}}{1-r_{k|k-1}^{\left(i\right)}}\langle p_{k|k-1}^{\left(i\right)},\psi_{k,z}\rangle },$$

with

(14) $$\psi_{k,z}\left(x\right)=g_{k}\left(z|x\right)p_{D,k}\left(x\right),$$

where $g_{k}\left(\cdot |x\right)$ is the measurement likelihood function, and $\kappa_{k}\left(\cdot \right)$ is the clutter intensity.

## 3. Extension of the Cardinality Balanced Multi-Target Multi-Bernoulli Filter

In the standard CBMeMBer filter of Section 2, both the target birth probability $r_{\Gamma ,k}^{\left(i\right)}$ and the target birth density $p_{\Gamma ,k}^{\left(i\right)}$ in Equation (5) are a priori assumed to be constant and independent of the measurements. However, the prior information about newborn targets is difficult to be obtained since the targets always appear irregularly over time in practice; for example, in monitoring, targets may appear from anywhere in the observation region at any time step, which makes the crude birth density and probability models invalid. In recent years, some investigations were developed for modeling the birth density [11,12,13,14,15], while the birth probability is still simply set as constant. This section firstly proposes a CBMeMBer filter with a completely measurement-driven target birth intensity, and then presents its SMC implementation.

### 3.1. CBMeMBer Filter Using Adaptive Target Birth Intensity

**Figure 1:**
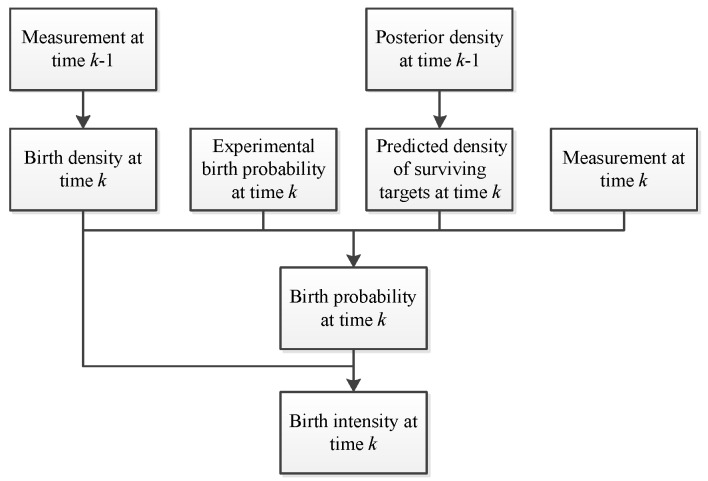
Modeling of the adaptive target birth intensity.

This subsection presents a new target birth intensity adaptively modeling both the target birth density [13] and the target birth probability. Figure 1 shows the framework of the adaptive target birth intensity modeling. The derivation of the improved adaptive target birth intensity CBMeMBer filter is presented below.

**(1) Target birth intensity modeling**

Similar to Reference [13], at each filtering step, the target birth density $p_{\Gamma ,k}^{\left(i\right)},i=1,\ldots ,M_{\Gamma ,k}$ is generated using measurements of the previous time step; thus, the target birth intensity ${\left\{\left(r_{\Gamma ,k}^{\left(i\right)},p_{\Gamma ,k}^{\left(i\right)}\right)\right\}}_{i=1}^{M_{\Gamma ,k}}$ can be re-expressed by

(15) $$\pi_{\Gamma ,k}={\left\{\left(r_{\Gamma ,k-1}^{\left(i\right)},p_{\Gamma ,k-1}^{\left(i\right)}\left(x|z_{i}\right)\right)\right\}}_{i=1}^{\left|Z_{k-1}\right|},$$

where the target birth probability $r_{\Gamma ,k-1}^{\left(i\right)}$ is assigned equally by $B_{\Gamma ,k}/\left|Z_{k-1}\right|$, with $B_{\Gamma ,k}$ being the expected number of target births and is usually set as a constant. This coarse determination of the expected number of newborn targets and the target birth probability is not always satisfied and can be problematic in certain cases. The birth probability that can adapt the background is quite demanding when capturing a real case. Below, a measurement-driven pre-processing method is proposed to properly adapt the real variation of the number of newborn targets.

Based on the target birth density in Equation (15), which removes the requirement for the prior position information of newborn targets, the corresponding target birth probability $r_{\Gamma ,k-1}^{\left(i\right)}$ is re-calculated using the current measurements instead of being uniformly assigned as $B_{\Gamma ,k}/\left|Z_{k-1}\right|$.

Suppose that, at time k−1, the posterior density of the CBMeMBer filter is a multi-Bernoulli distribution with

(16) $$\pi_{k-1}={\left\{\left(r_{k-1}^{\left(i\right)},p_{k-1}^{\left(i\right)}\right)\right\}}_{i=1}^{M_{k-1}}.$$

**Proposition** **1.** *In the pre-processing step, an experimental expectation ${\hat{B}}_{\Gamma ,k}$ on newborn target number, e.g., ${\hat{B}}_{\Gamma ,k}=0.2$, is assumed and assigned to each target birth density with an identical probability ${\hat{r}}_{\Gamma ,k-1}^{\left(i\right)}={\hat{B}}_{\Gamma ,k}/\left|Z_{k-1}\right|$. Applying the standard CBMeMBer prediction step, the predicted multi-target density of surviving targets is obtained as*

(17) $$\pi_{P,k|k-1}={\left\{\left(r_{P,k|k-1}^{\left(i\right)},p_{P,k|k-1}^{\left(i\right)}\right)\right\}}_{i=1}^{M_{k-1}},$$

and the experimental target birth intensity,

(18) $${\hat{\pi }}_{\Gamma ,k}={\left\{\left({\hat{r}}_{\Gamma ,k-1}^{\left(i\right)},p_{\Gamma ,k-1}^{\left(i\right)}\left(x|z_{i}\right)\right)\right\}}_{i=1}^{\left|Z_{k-1}\right|},$$

can be seen as a surmise of the initiation of the newborn target tracks. By correcting the experimental target birth intensity ${\hat{\pi }}_{\Gamma ,k}$ through the prediction of the surviving targets and the current measurements $z^{\prime }\in Z_{k}$, a modified target birth probability ${\tilde{r}}_{\Gamma ,k-1}^{\left(i\right)},i=1,\ldots ,\left|Z_{k-1}\right|$ can be derived. Each of the modified target birth probabilities is calculated separately with full consideration of the detection of the new-born target, which can be given by

(19) $${\tilde{r}}_{\Gamma ,k-1}^{\left(i\right)}={\tilde{r}}_{L\Gamma ,k-1}^{\left(i\right)}+{\tilde{r}}_{U\Gamma ,k-1}^{\left(i\right)},$$

where ${\tilde{r}}_{L\Gamma ,k-1}^{\left(i\right)}$ and ${\tilde{r}}_{U\Gamma ,k-1}^{\left(i\right)}$ represent the modeling of the birth probability under newborn targets being missed and detected by sensors, respectively. ${\tilde{r}}_{L\Gamma ,k-1}^{\left(i\right)}$ can be expressed by

(20) $${\tilde{r}}_{L\Gamma ,k-1}^{\left(i\right)}={\hat{r}}_{\Gamma ,k-1}^{\left(i\right)}\frac{1-\langle p_{\Gamma ,k-1}^{\left(i\right)}\left(\cdot |z_{i}\right),p_{D,k}\rangle }{1-{\hat{r}}_{\Gamma ,k-1}^{\left(i\right)}\langle p_{\Gamma ,k-1}^{\left(i\right)}\left(\cdot |z_{i}\right),p_{D,k}\rangle },$$

which is derived similar to the legacy tracks in the standard CBMeMBer filter, and ${\tilde{r}}_{U\Gamma ,k-1}^{\left(i\right)}$ can be expressed by

(21) $${\tilde{r}}_{U\Gamma ,k-1}^{\left(i\right)}=\sum_{z^{\prime }\in Z_{k}}\frac{G_{k}\left(z^{\prime }\right)}{\kappa_{k}\left(z^{\prime }\right)+S_{\Gamma k}\left(z^{\prime }\right)+S_{Uk}\left(z^{\prime }\right)},$$

which involves operations traversing all the measurements, with

(22) $$G_{k}\left(z^{\prime }\right)=\frac{{\hat{r}}_{\Gamma ,k-1}^{\left(i\right)}\left(1-{\hat{r}}_{\Gamma ,k-1}^{\left(i\right)}\right)\langle p_{\Gamma ,k-1}^{\left(i\right)}\left(\cdot |z_{i}\right),\psi_{k,z^{\prime }}\rangle }{{\left(1-{\hat{r}}_{\Gamma ,k-1}^{\left(i\right)}\langle p_{\Gamma ,k-1}^{\left(i\right)}\left(\cdot |z_{i}\right),p_{D,k}\rangle \right)}^{2}},$$

(23) $$S_{\Gamma k}\left(z^{\prime }\right)=\sum_{i=1}^{\left|Z_{k-1}\right|}\frac{{\hat{r}}_{\Gamma ,k-1}^{\left(i\right)}\langle p_{\Gamma ,k-1}^{\left(i\right)}\left(\cdot |z_{i}\right),\psi_{k,z^{\prime }}\rangle }{1-{\hat{r}}_{\Gamma ,k-1}^{\left(i\right)}\langle p_{\Gamma ,k-1}^{\left(i\right)}\left(\cdot |z_{i}\right),p_{D,k}\rangle },$$

(24) $$S_{Uk}\left(z^{\prime }\right)=\sum_{i=1}^{M_{k-1}}\frac{r_{P,k|k-1}^{\left(i\right)}\langle p_{P,k|k-1}^{\left(i\right)},\psi_{k,z^{\prime }}\rangle }{1-r_{P,k|k-1}^{\left(i\right)}\langle p_{P,k|k-1}^{\left(i\right)},p_{D,k}\rangle }.$$

$G_{k}\left(\cdot \right)$ can be seen as a function that reflects the correlation between the birth component and the measurements. $S_{\Gamma k}\left(\cdot \right)$ and $S_{Uk}\left(\cdot \right)$ can be seen as normalization factors. With Equations (19)–(24), the modified target birth probability ${\tilde{r}}_{\Gamma ,k-1}^{\left(i\right)}$ can be obtained, and explained as a rational assumption of the average number of newborn targets appearing in the corresponding birth area.

However, ${\tilde{r}}_{\Gamma ,k-1}^{\left(i\right)}$ may be overestimated due to the clutter or the surviving targets, which can be mitigated by a hard-limited upper bound $r_{\max}$. As a result, the required target birth probability $r_{\Gamma ,k-1}^{\left(i\right)}$ can be obtained as

(25) $$r_{\Gamma ,k-1}^{\left(i\right)}=\min\left({\tilde{r}}_{\Gamma ,k-1}^{\left(i\right)},r_{\max}\right),i=1,\ldots ,\left|Z_{k-1}\right|.$$

Table 1 summarizes the modeling of the target birth probability via pseudo code.

Next, we integrate the improved birth probability into the normal filtering procedure.

**(2) Prediction**

Upon substituting the experimental target birth probability ${\hat{r}}_{\Gamma ,k-1}^{\left(i\right)},i=1,\ldots ,\left|Z_{k-1}\right|$ with $r_{\Gamma ,k-1}^{\left(i\right)},i=1,\ldots ,\left|Z_{k-1}\right|$, the new target birth intensity model ${\left\{\left(r_{\Gamma ,k-1}^{\left(i\right)},p_{\Gamma ,k-1}^{\left(i\right)}\left(\cdot |z_{i}\right)\right)\right\}}_{i=1}^{\left|Z_{k-1}\right|}$ can be obtained and applied in the subsequent filtering step. Since the predicted density of surviving targets $\pi_{P,k|k-1}$ is irrelevant to that of newborn targets and was computed in the pre-processing step, the re-calculation of the predicted surviving target density can be avoided in the normal filtering. Thus, the predicted density can be presented by

(26) $$\pi_{k|k-1}={\left\{\left(r_{\Gamma ,k-1}^{\left(i\right)},p_{\Gamma ,k-1}^{\left(i\right)}\left(x|z_{i}\right)\right)\right\}}_{i=1}^{\left|Z_{k-1}\right|}\cup {\left\{\left(r_{P,k|k-1}^{\left(i\right)},p_{P,k|k-1}^{\left(i\right)}\right)\right\}}_{i=1}^{M_{k-1}},$$

and can be further expressed as Equation (8) with $M_{k|k-1}=\left|Z_{k-1}\right|+M_{k-1}$.

**(3) Update**

Following that, the normal update step proceeds similarly to the standard CBMeMBer filter, with the new adaptive target birth intensity model involved. In updating, the calculation of the normalization function in Equation (24) can be preserved and directly imported when it is needed into Equation (12). As a result, the posterior density is obtained and the full recursion of time k is completed.

**Remark** **1.** *Note that the target birth density is determined by the measurements of previous time steps; thus, misrecognition of clutter can be avoided. Moreover, the target birth probability is calculated in the pre-processing step without prior information requirements of the number of newborn targets. As a result, compared to the CBMeMBer filter that only adopts the adaptive target birth density, the proposed filter can achieve a better tracking accuracy.*

### 3.2. Implementation

This subsection presents the SMC implementation of the proposed CBMeMBer filter. The SMC implementation uses particle sets to approximate the densities in filters, which relieves the restriction of linearity and Gaussian assumptions.

**(1) Target birth intensity modeling**

At time k−1, the posterior density of the target is given by Equation (16), where

(27) $$p_{k-1}^{\left(i\right)}\left(x\right)=\sum_{j=1}^{L_{k-1}^{\left(i\right)}}w_{k-1}^{\left(i,j\right)}\delta_{x_{k-1}^{\left(i,j\right)}}\left(x\right).$$

The probability density $p_{k-1}^{\left(i\right)}\left(x\right)$ is expressed by the weighted particle set ${\left\{w_{k-1}^{\left(i,j\right)},x_{k-1}^{\left(i,j\right)}\right\}}_{j=1}^{L_{k-1}^{\left(i\right)}}$, where $L_{k-1}^{\left(i\right)}$ is the number of particles.

**Target birth density:** The target birth density $p_{\Gamma ,k-1}^{\left(i\right)}\left(\cdot |z_{i}\right)$ of the newborn target intensity ${\left\{\left(r_{\Gamma ,k-1}^{\left(i\right)},p_{\Gamma ,k-1}^{\left(i\right)}\left(\cdot |z_{i}\right)\right)\right\}}_{i=1}^{\left|Z_{k-1}\right|}$ is expressed by a weighted particle set $x_{\Gamma ,k-1}^{\left(i,j\right)},j=1,\ldots ,L_{\Gamma ,k-1}^{\left(i\right)}$, generated from measurement $z_{i}\in Z_{k-1}$. Note that the target state can be written as $x={\left[p^{T},v^{T}\right]}^{T}$, where p is the component measured by the sensor, and v is the unmeasured component. Therefore, the measurement can be written as $z=h^{\prime }\left(p\right)+\varepsilon$, where ε is a Gaussian measurement noise with zero mean and covariance R. Particles $p^{\left(j\right)}$ can be obtained from $N\left(p;{h^{\prime }}^{-1}\left(z\right),HRH^{T}\right)$, where H is the Jacobi matrix of ${h^{\prime }}^{-1}$, and particles $v^{\left(j\right)}$ from unmeasured subspace are set a priori. The weight of each particle is identical, i.e., $w_{\Gamma ,k-1}^{\left(i,j\right)}=\frac{1}{L_{\Gamma ,k-1}^{\left(i\right)}}$. Consequently, the target birth density can be expressed as

(28) $$p_{\Gamma ,k-1}^{\left(i\right)}\left(x|z_{i}\right)=\sum_{j=1}^{L_{\Gamma ,k-1}^{\left(i\right)}}w_{\Gamma ,k-1}^{\left(i,j\right)}\delta_{x_{\Gamma ,k-1}^{\left(i,j\right)}}\left(x\right).$$

**Target birth probability:** The target birth probability $r_{\Gamma ,k-1}^{\left(i\right)}$ of the newborn target intensity ${\left\{\left(r_{\Gamma ,k-1}^{\left(i\right)},p_{\Gamma ,k-1}^{\left(i\right)}\left(\cdot |z_{i}\right)\right)\right\}}_{i=1}^{\left|Z_{k-1}\right|}$ is calculated by a pre-processing step. The predicted density of surviving targets, ${\left\{\left(r_{P,k|k-1}^{\left(i\right)},p_{P,k|k-1}^{\left(i\right)}\right)\right\}}_{i=1}^{M_{k-1}}$, can be computed as follows:

(29) $$r_{P,k|k-1}^{\left(i\right)}=r_{k-1}^{\left(i\right)}\sum_{j=1}^{L_{k-1}^{\left(i\right)}}w_{k-1}^{\left(i,j\right)}p_{S,k}\left(x_{k-1}^{\left(i,j\right)}\right),$$

(30) $$p_{P,k|k-1}^{\left(i\right)}\left(x\right)=\sum_{j=1}^{L_{k-1}^{\left(i\right)}}w_{P,k|k-1}^{\left(i,j\right)}\delta_{x_{P,k|k-1}^{\left(i,j\right)}}\left(x\right),$$

where

(31) $$x_{P,k|k-1}^{\left(i,j\right)}∼q_{k}^{\left(i\right)}\left(\cdot |x_{k-1}^{\left(i,j\right)},Z_{k}\right),j=1,\ldots ,L_{k-1}^{\left(i\right)},$$

(32) $$w_{P,k|k-1}^{\left(i,j\right)}=\frac{w_{k-1}^{\left(i,j\right)}f_{k|k-1}\left(x_{P,k|k-1}^{\left(i,j\right)}|x_{k-1}^{\left(i,j\right)}\right)p_{S,k}\left(x_{k-1}^{\left(i,j\right)}\right)}{q_{k}^{\left(i\right)}\left(x_{P,k|k-1}^{\left(i,j\right)}|x_{k-1}^{\left(i,j\right)},Z_{k}\right)}/\sum_{j=1}^{L_{k-1}^{\left(i\right)}}w_{k-1}^{\left(i,j\right)}p_{S,k}\left(x_{k-1}^{\left(i,j\right)}\right),$$

and $q_{k}^{\left(i\right)}\left(\cdot |x_{k-1}^{\left(i,j\right)},Z_{k}\right)$ represents the importance density. To simplify the SMC implementation, it is obtained that $q_{k}^{\left(i\right)}\left(\cdot |x_{k-1}^{\left(i,j\right)},Z_{k}\right)=f_{k|k-1}\left(\cdot |x_{k-1}^{\left(i,j\right)}\right)$.

The modified target birth probability ${\tilde{r}}_{\Gamma ,k-1}^{\left(i\right)}$ is calculated by Equation (19), where ${\tilde{r}}_{L\Gamma ,k-1}^{\left(i\right)}$ is given by

(33) $${\tilde{r}}_{L\Gamma ,k-1}^{\left(i\right)}={\hat{r}}_{\Gamma ,k-1}^{\left(i\right)}\frac{1-\sum_{j=1}^{L_{\Gamma ,k-1}^{\left(i\right)}}w_{\Gamma ,k-1}^{\left(i,j\right)}p_{D,k}\left(x_{\Gamma ,k-1}^{\left(i,j\right)}\right)}{1-{\hat{r}}_{\Gamma ,k-1}^{\left(i\right)}\sum_{j=1}^{L_{\Gamma ,k-1}^{\left(i\right)}}w_{\Gamma ,k-1}^{\left(i,j\right)}p_{D,k}\left(x_{\Gamma ,k-1}^{\left(j\right)}\right)},$$

and ${\tilde{r}}_{U\Gamma ,k-1}\left(z\right)$ can be written as Equation (21), where

(34) $$G_{k}\left(z^{\prime }\right)=\frac{{\hat{r}}_{\Gamma ,k-1}^{\left(i\right)}(1-{\hat{r}}_{\Gamma ,k-1}^{\left(i\right)})(1-{\hat{r}}_{\Gamma ,k-1}^{\left(i\right)})\sum_{j=1}^{L_{\Gamma ,k-1}^{\left(i\right)}}w_{\Gamma ,k-1}^{\left(i,j\right)}\psi_{k,z^{\prime }}(x_{\Gamma ,k-1}^{\left(i,j\right)})}{{\left(1-{\hat{r}}_{\Gamma ,k-1}^{\left(i\right)}\sum_{j=1}^{L_{\Gamma ,k-1}^{\left(i\right)}}w_{\Gamma ,k-1}^{\left(i,j\right)}p_{D,k}(x_{\Gamma ,k-1}^{\left(i,j\right)})\right)}^{2}},$$

(35) $$S_{\Gamma k}\left(z^{\prime }\right)=\sum_{i=1}^{\left|Z_{k-1}\right|}\frac{{\hat{r}}_{\Gamma ,k-1}^{\left(i\right)}\sum_{j=1}^{L_{\Gamma ,k-1}^{\left(i\right)}}w_{\Gamma ,k-1}^{\left(i,j\right)}\psi_{k,z^{\prime }}(x_{\Gamma ,k-1}^{\left(i,j\right)})}{1-{\hat{r}}_{\Gamma ,k-1}^{\left(i\right)}\sum_{j=1}^{L_{\Gamma ,k-1}^{\left(i\right)}}w_{\Gamma ,k-1}^{\left(i,j\right)}p_{D,k}(x_{\Gamma ,k-1}^{\left(i,j\right)})},$$

(36) $$S_{Uk}\left(z^{\prime }\right)=\sum_{i=1}^{M_{k-1}}\frac{{\hat{r}}_{P,k|k-1}^{\left(i\right)}\sum_{j=1}^{L_{k-1}^{\left(i\right)}}w_{P,k|k-1}^{\left(i,j\right)}\psi_{k,z^{\prime }}(x_{P,k|k-1}^{\left(i,j\right)})}{1-{\hat{r}}_{P,k|,k-1}^{\left(i\right)}\sum_{j=1}^{L_{k-1}^{\left(i\right)}}w_{P,k|,k-1}^{\left(i,j\right)}p_{D,k}(x_{P,k|,k-1}^{\left(i,j\right)})}.$$

Then, the target birth probability $r_{\Gamma ,k-1}^{\left(i\right)}$ can be obtained using Equation (25).

**(2) Prediction**

In the normal filtering step, the predicted multi-target density, integrating ${\left\{\left(r_{\Gamma ,k-1}^{\left(i\right)},p_{\Gamma ,k-1}^{\left(i\right)}\left(\cdot |z_{i}\right)\right)\right\}}_{i=1}^{\left|Z_{k-1}\right|}$ and ${\left\{\left(r_{P,k|k-1}^{\left(i\right)},p_{P,k|k-1}^{\left(i\right)}\right)\right\}}_{i=1}^{M_{k-1}}$, can be expressed by ${\left\{\left(r_{k|k-1}^{\left(i\right)},p_{k|k-1}^{\left(i\right)}\right)\right\}}_{i=1}^{M_{k|k-1}}$, where

(37) $$p_{k|k-1}^{\left(i\right)}\left(x\right)=\sum_{j=1}^{L_{k|k-1}^{\left(i\right)}}w_{k|k-1}^{\left(i,j\right)}\delta_{x_{k|k-1}^{\left(i,j\right)}}\left(x\right).$$

**(3) Update**

The updated posterior multi-target density can be expressed in the same form as Equation (9) and is computed as follows:

(38) $$r_{L,k}^{\left(i\right)}=r_{k|k-1}^{\left(i\right)}\frac{1-\alpha_{L,k}^{\left(i\right)}}{1-r_{k|k-1}^{\left(i\right)}\alpha_{L,k}^{\left(i\right)}},$$

(39) $$p_{L,k}^{\left(i\right)}\left(x\right)=\sum_{j=1}^{L_{k|k-1}^{\left(i\right)}}w_{L,k}^{\left(i,j\right)}\delta_{x_{k|k-1}^{\left(i,j\right)}}\left(x\right),$$

(40) $$r_{U,k}\left(z\right)=\frac{\sum_{i=1}^{M_{k|k-1}}\frac{r_{k|k-1}^{\left(i\right)}\left(1-r_{k|k-1}^{\left(i\right)}\right)\alpha_{U,k}^{\left(i\right)}\left(z\right)}{{\left(1-r_{k|k-1}^{\left(i\right)}\alpha_{L,k}^{\left(i\right)}\right)}^{2}}}{\kappa_{k}\left(z\right)+\sum_{i=1}^{M_{k|k-1}}\frac{r_{k|k-1}^{\left(i\right)}\alpha_{U,k}^{\left(i\right)}\left(z\right)}{1-r_{k|k-1}^{\left(i\right)}\alpha_{L,k}^{\left(i\right)}}},$$

(41) $$p_{U,k}\left(x;z\right)=\sum_{i=1}^{M_{k|k-1}}\sum_{j=1}^{L_{k|k-1}^{\left(i\right)}}w_{U,k}^{\left(i,j\right)}\left(z\right)\delta_{x_{k|k-1}^{\left(i,j\right)}}\left(x\right),$$

where

(42) $$\alpha_{L,k}^{\left(i\right)}=\sum_{j=1}^{L_{k|k-1}^{\left(i\right)}}w_{k|k-1}^{\left(i,j\right)}p_{D,k}\left(x_{k|k-1}^{\left(i,j\right)}\right),$$

(43) $$w_{L,k}^{\left(i,j\right)}=w_{k|k-1}^{\left(i,j\right)}\left(1-p_{D,k}\left(x_{k|k-1}^{\left(i,j\right)}\right)\right)/\sum_{j=1}^{L_{k|k-1}^{\left(i\right)}}w_{k|k-1}^{\left(i,j\right)}\left(1-p_{D,k}\left(x_{k|k-1}^{\left(i,j\right)}\right)\right),$$

(44) $$\alpha_{U,k}^{\left(i\right)}\left(z\right)=\sum_{j=1}^{L_{k|k-1}^{\left(i\right)}}w_{k|k-1}^{\left(i,j\right)}\psi_{k,z}\left(x_{k|k-1}^{\left(i,j\right)}\right),$$

(45) $$w_{U,k}^{\left(i,j\right)}\left(z\right)=\frac{w_{k|k-1}^{\left(i,j\right)}\frac{r_{k|k-1}^{\left(i\right)}}{1-r_{k|k-1}^{\left(i\right)}}\psi_{k,z}\left(x_{k|k-1}^{\left(i,j\right)}\right)}{\sum_{i=1}^{M_{k|k-1}}\sum_{j=1}^{L_{k|k-1}^{\left(i\right)}}w_{k|k-1}^{\left(i,j\right)}\frac{r_{k|k-1}^{\left(i\right)}}{1-r_{k|k-1}^{\left(i\right)}}\psi_{k,z}\left(x_{k|k-1}^{\left(i,j\right)}\right)}.$$

**Remark** **2.** *For the SMC implementation, weight degeneration is unavoidable. To mitigate the effect of degeneration, a resampling method is applied to the tracks after the update step. The number of targets is estimated by the cardinality mean, and the states of targets are estimated by computing the means of the posterior probability densities with comparatively high existence probabilities in $\pi_{k}$.*

## 4. Fast Sequential Monte Carlo Cardinality Balanced Multi-Target Multi-Bernoulli Filter with Adaptive Target Birth Intensity

**Figure 2:**
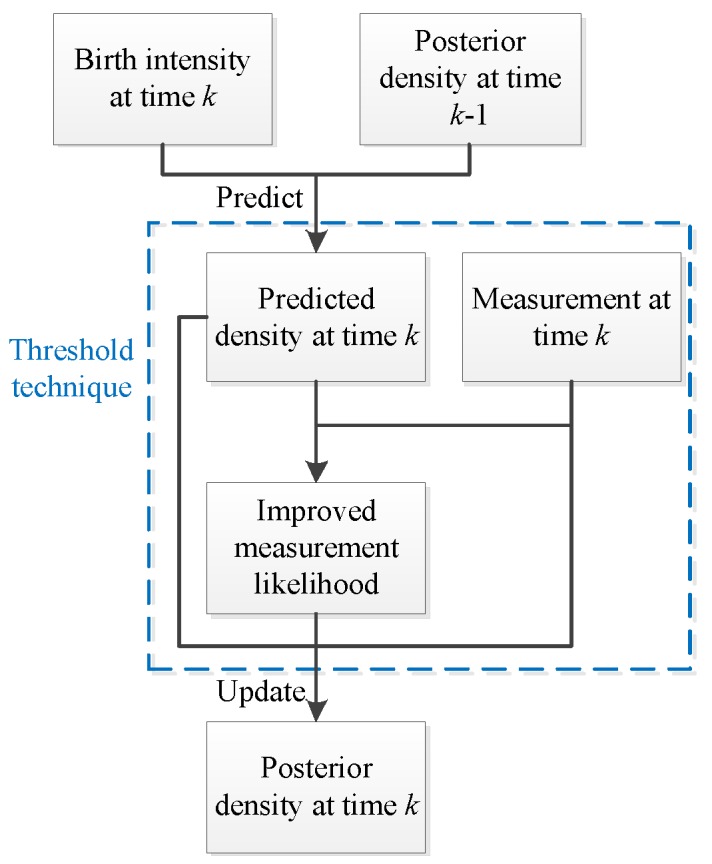
Cardinality balanced multi-target multi-Bernoulli (CBMeMBer) filter with threshold technique.

In this section, a threshold method used in the calculation of the measurement likelihoods is proposed and then applied to the SMC CBMeMBer filter presented in Section 3. Figure 2 shows a framework of the CBMeMBer filter integrating the threshold technique.

### 4.1. Improved Measurement Likelihood

In general, the proposed CBMeMBer filter with adaptive target birth intensity has a heavy computational burden compared to the standard CBMeMBer filter. This is because the target birth density is modeled by the measurement, and the target birth probability is modeled by a pre-processing step, which requires more calculation in both the birth modeling step and the subsequent update step. The SMC implementation of this filter is notably time-consuming, especially for the cases with a large number of particles to describe the target states or a large number of measurements.

To reduce the computational burden, an improved measurement likelihood is proposed. In MTT, the measurement likelihood is generally computed by

(46) $$l_{k}\left(x|z\right)≜g_{k}\left(z|x\right).$$

Then, a pre-determination can be used to evaluate whether this measurement likelihood is worth being preserved or not.

**Proposition** **2.** *For each target state x, there exists a mapping on observation space based on the measurement function, i.e.,*

(47) $$\overset{⌣}{z}=h\left(x\right).$$

If the mapping $\overset{⌣}{z}$ is far from the measurement z, the corresponding target state is rarely to be matched with the measurement, and the likelihood between them will be a small value, i.e., such a target state contributes little to the filters. Hence, we can directly set this likelihood as zero to avoid extra calculations. Below, a threshold is introduced to measure the distance between the measurement and the mapping of the target. The improved measurement likelihood can be expressed as

(48) $${\tilde{l}}_{k}\left(x|z\right)=\{\begin{array}{cc} g_{k}(z|x), & if\;D\left(\overset{⌣}{z},z\right)\leq U\\ 0, & otherwise \end{array},$$

where U represents the threshold and $D\left(\overset{⌣}{z},z\right)$ represents the distance between $\overset{⌣}{z}$ and z. Note that the mapping $\overset{⌣}{z}={\left[{\overset{⌣}{z}}_{1},\ldots ,{\overset{⌣}{z}}_{n}\right]}^{T}={\left[h{\left(x\right)}_{1},\ldots ,h{\left(x\right)}_{n}\right]}^{T}$ and the measurement $z={\left[z_{1},\ldots ,z_{n}\right]}^{T}$ are both multi-dimensional, where n is the dimension of the measurement space. Thus, the distance $D\left(\overset{⌣}{z},z\right)$ and the threshold U are both vectors, and the pre-determination condition in Equation (48) can be fully formulated as

(49) $$D\left(\overset{⌣}{z},z\right)\leq U\Leftrightarrow \{\begin{array}{c} d\left({\overset{⌣}{z}}_{1},z_{1}\right)\leq u_{1}\\ \ldots \\ d\left({\overset{⌣}{z}}_{n},z_{n}\right)\leq u_{n} \end{array},$$

where $d\left({\overset{⌣}{z}}_{i},z_{i}\right),\;i=1,\ldots ,n$ represents the distance, such as Euclidean distance, between the corresponding elements of the mapping $\overset{⌣}{z}$ and the measurement z, and $u_{i},\;i=1,\ldots ,n$ represents the corresponding threshold.

### 4.2. Threshold Selection

In the improved measurement likelihood, the unnecessary calculation is recognized and removed through a threshold U. A suitable threshold should be a bridge which can properly reflect the correlation between the likelihood $g_{k}\left(z|x\right)$ and the distance $D\left(\overset{⌣}{z},z\right)$. Here, we take the measurement noise as the basic part of the threshold selection.

**Proposition** **3.** *Suppose that the measurement noise follows a zero-mean Gaussian distribution with covariance matrix*

(50) $$R=diag\left({\left[\sigma_{1}^{2},\ldots ,\sigma_{n}^{2}\right]}^{T}\right),$$

where $\sigma_{i},\;i=1,\ldots ,n$ represents the standard deviation. The threshold $U=\left[u_{1},\ldots ,u_{n}\right]$ can be set as $\left[\beta_{1}\times \sigma_{1},\ldots ,\beta_{n}\times \sigma_{n}\right]$, where $\beta_{i},\;i=1,\ldots ,n$ represents a non-negative scale parameter. The scale parameter can be seen as a coefficient of the standard deviation $\sigma_{i}$ and can be used to zoom the threshold element $u_{1}$. For simplification, we set the scale parameters to be identical, i.e., $\beta_{1}=\ldots =\beta_{n}=\beta$. Thus, the threshold element can be replaced by

(51) $$u_{i}=\beta \times \sigma_{i},\;i=1,\ldots ,n.$$

Obviously, under known measurement function and noise, the probability of reserving the useful information can be easily worked out based on the rule of thumb, which can be expressed by

(52) $$\theta_{\beta }=P\left(d\left({\overset{⌣}{z}}_{i},z_{i}\right)\leq \beta \times \sigma_{i}\right),\;i=1,\ldots ,n,$$

where $P\left(d\left({\overset{⌣}{z}}_{i},z_{i}\right)\leq \beta \times \sigma_{i}\right)$ represents the probability that the distance getting a value does not exceed the threshold in the ith dimension of the observation space. Through the cumulative distribution function (CDF) of the measurement noise, the probability $\theta_{\beta }$ can be easily obtained. For instance, by assuming zero-mean Gaussian measurement noise, we obtain $\theta_{\beta =1}\approx 68.27\%$, $\theta_{\beta =2}\approx 95.45\%$, $\theta_{\beta =3}\approx 99.73\%$, and $\theta_{\beta =4}\approx 99.99\%$. On the other hand, if there exists an expectation of the probability $\theta_{\beta }$, e.g., $\theta_{\beta }=98\%$, the appropriate scale parameter β can be easily calculated by applying the inverse operation of the CDF.

**Proposition** **4.** *To correctly integrate the proposed measurement likelihood into the Bayesian based MTT systems, the clutter density* $\kappa_{k}\left(z\right)$ *used in updating should be adjusted. For the common uniform distribution clutter model, the adjusted clutter density can be expressed by*

(53) $${\tilde{\kappa }}_{k}\left(z\right)=\theta_{\beta }\times \kappa_{k}\left(z\right).$$

It shows that only the main factor from the clutter is considered, with a proportion $\theta_{\beta }$ that is consistent with the improved measurement likelihood. In the CBMeMBer filter, the update step using the new measurement of likelihood can be rewritten as

(54) $$r_{U,k}\left(z\right)=\frac{\sum_{i=1}^{M_{k|k-1}}\frac{r_{k|k-1}^{\left(i\right)}\left(1-r_{k|k-1}^{\left(i\right)}\right)\langle p_{k|k-1}^{\left(i\right)},{\tilde{\psi }}_{k,z}\rangle }{{\left(1-r_{k|k-1}^{\left(i\right)}\langle p_{k|k-1}^{\left(i\right)},p_{D,k}\rangle \right)}^{2}}}{{\tilde{\kappa }}_{k}\left(z\right)+\sum_{i=1}^{M_{k|k-1}}\frac{r_{k|k-1}^{\left(i\right)}\langle p_{k|k-1}^{\left(i\right)},{\tilde{\psi }}_{k,z}\rangle }{1-r_{k|k-1}^{\left(i\right)}\langle p_{k|k-1}^{\left(i\right)},p_{D,k}\rangle }},$$

(55) $$p_{U,k}\left(x;z\right)=\frac{\sum_{i=1}^{M_{k|k-1}}\frac{r_{k|k-1}^{\left(i\right)}}{1-r_{k|k-1}^{\left(i\right)}}p_{k|k-1}^{\left(i\right)}\left(x\right){\tilde{\psi }}_{k,z}\left(x\right)}{\sum_{i=1}^{M_{k|k-1}}\frac{r_{k|k-1}^{\left(i\right)}}{1-r_{k|k-1}^{\left(i\right)}}\langle p_{k|k-1}^{\left(i\right)},{\tilde{\psi }}_{k,z}\rangle },$$

where

(56) $${\tilde{\psi }}_{k,z}\left(x\right)={\tilde{l}}_{k}\left(x|z\right)p_{D,k}\left(x\right).$$

**Remark** **3.** *The target states updated in this step evolve from a prediction step, which is biased to the true target states. Thus, the proportion of the reserved contributions from targets cannot be exactly measured by*$\theta_{\beta }$*. Under general tracking scenarios, considering that the gap between the predicted and the true target states is seldom extremely large, the probability*$\theta_{\beta }$*can still be a reference in choosing the scale parameter*β.

### 4.3. Fast Sequential Monte Carlo Adaptive Target Birth Intensity Cardinality Balanced Multi-Target Multi-Bernoulli Filter

In this subsection, the threshold method is applied to our proposed SMC CBMeMBer filter to relieve the huge computational burden by reducing the likelihood calculation. The new threshold SMC CBMeMBer filter is called the fast adaptive target birth intensity SMC CBMeMBer filter.

**Proposition** **5.** *In the fast filter, the threshold is applied in both the pre-processing step and the update step. By substituting the clutter density*$\kappa_{k}\left(z\right)$*with*${\tilde{\kappa }}_{k}\left(z\right)$*and the measurement likelihood*$l_{k}\left(x|z\right)$*with*${\tilde{l}}_{k}\left(x|z\right)$*, Equation (21) in the pre-processing step can be rewritten as*

(57) $${\tilde{r}}_{U\Gamma ,k-1}^{\left(i\right)}=\sum_{z^{\prime }\in Z_{k}}\frac{{\tilde{G}}_{k}\left(z^{\prime }\right)}{{\tilde{\kappa }}_{k}\left(z^{\prime }\right)+{\tilde{S}}_{\Gamma k}\left(z^{\prime }\right)+{\tilde{S}}_{Uk}\left(z^{\prime }\right)},$$

where

(58) $${\tilde{G}}_{k}\left(z^{\prime }\right)=\frac{{\hat{r}}_{\Gamma ,k-1}^{\left(i\right)}(1-{\hat{r}}_{\Gamma ,k-1}^{\left(i\right)})\sum_{j=1}^{L_{\Gamma ,k-1}^{\left(i\right)}}w_{\Gamma ,k-1}^{\left(i,j\right)}{\tilde{\psi }}_{k,z^{\prime }}(x_{\Gamma ,k-1}^{\left(i,j\right)})}{{\left(1-{\hat{r}}_{\Gamma ,k-1}^{\left(i\right)}\sum_{j=1}^{L_{\Gamma ,k-1}^{\left(i\right)}}w_{\Gamma ,k-1}^{\left(i,j\right)}p_{D,k}(x_{\Gamma ,k-1}^{\left(i,j\right)})\right)}^{2}},$$

(59) $${\tilde{S}}_{\Gamma k}\left(z^{\prime }\right)=\sum_{i=1}^{\left|Z_{k-1}\right|}\frac{{\hat{r}}_{\Gamma ,k-1}^{\left(i\right)}\sum_{j=1}^{L_{\Gamma ,k-1}^{\left(i\right)}}w_{\Gamma ,k-1}^{\left(i,j\right)}{\tilde{\psi }}_{k,z^{\prime }}(x_{\Gamma ,k-1}^{\left(i,j\right)})}{1-{\hat{r}}_{\Gamma ,k-1}^{\left(i\right)}\sum_{j=1}^{L_{\Gamma ,k-1}^{\left(i\right)}}w_{\Gamma ,k-1}^{\left(i,j\right)}p_{D,k}(x_{\Gamma ,k-1}^{\left(i,j\right)})},$$

(60) $${\tilde{S}}_{Uk}\left(z^{\prime }\right)=\sum_{i=1}^{M_{k-1}}\frac{r_{P,k|k-1}^{\left(i\right)}\sum_{j=1}^{L_{k-1}^{\left(i\right)}}w_{P,k|k-1}^{\left(i,j\right)}{\tilde{\psi }}_{k,z^{\prime }}(x_{P,k|k-1}^{\left(i,j\right)})}{1-r_{P,k|k-1}^{\left(i\right)}\sum_{j=1}^{L_{k-1}^{\left(i\right)}}w_{P,k|k-1}^{\left(i,j\right)}p_{D,k}(x_{P,k|k-1}^{\left(i,j\right)})}.$$

Equation (40) in the update step can be rewritten as

(61) $$r_{U,k}\left(z\right)=\frac{\sum_{i=1}^{M_{k|k-1}}\frac{r_{k|k-1}^{\left(i\right)}\left(1-r_{k|k-1}^{\left(i\right)}\right){\tilde{\alpha }}_{U,k}^{\left(i\right)}\left(z\right)}{{\left(1-r_{k|k-1}^{\left(i\right)}\alpha_{L,k}^{\left(i\right)}\right)}^{2}}}{{\tilde{\kappa }}_{k}\left(z\right)+\sum_{i=1}^{M_{k|k-1}}\frac{r_{k|k-1}^{\left(i\right)}{\tilde{\alpha }}_{U,k}^{\left(i\right)}\left(z\right)}{1-r_{k|k-1}^{\left(i\right)}\alpha_{L,k}^{\left(i\right)}}},$$

where

(62) $${\tilde{\alpha }}_{U,k}^{\left(i\right)}(z)=\sum_{j=1}^{L_{k|k-1}^{\left(i\right)}}w_{k|k-1}^{\left(i,j\right)}{\tilde{\psi }}_{k,z}\left(x_{k|k-1}^{\left(i,j\right)}\right).$$

Equation (45) can be rewritten as

(63) $$w_{U,k}^{\left(i,j\right)}\left(z\right)=\frac{w_{k|k-1}^{\left(i,j\right)}\frac{r_{k|k-1}^{\left(i\right)}}{1-r_{k|k-1}^{\left(i\right)}}{\tilde{\psi }}_{k,z}\left(x_{k|k-1}^{\left(i,j\right)}\right)}{\sum_{i=1}^{M_{k|k-1}}\sum_{j=1}^{L_{k|k-1}^{\left(i\right)}}w_{k|k-1}^{\left(i,j\right)}\frac{r_{k|k-1}^{\left(i\right)}}{1-r_{k|k-1}^{\left(i\right)}}{\tilde{\psi }}_{k,z}\left(x_{k|k-1}^{\left(i,j\right)}\right)}.$$

**Remark** **4.** *Suppose that the size of the observation region in each dimension is represented by* $\Lambda_{i},i=1,\ldots ,n$ *; since the clutter follows a uniform distribution, the measurement likelihood calculation from the clutter can be cut down by*

(64) $$\left(1-\prod_{i=1}^{n}\frac{2u_{i}}{\Lambda_{i}}\right)\times 100\%.$$

The percentage can be very high since the size of the observation region is much larger than that of the threshold based on the measurement noise, i.e., $\Lambda_{i}\gg 2u_{i},\;i=1,\ldots ,n$.

**Remark** **5.** *Note that the fast CBMeMBer filter updates the predicted density only considering the nearby measurements and, thus, avoids a large number of unnecessary or even harmful calculations from the long-distance clutters. It can greatly reduce the negative impact of the clutters to the filter and may improve the target tracking accuracy. It is worth noting that there exists a trade-off between letting the true measurement in and keeping the clutters out. In general, the threshold will be proper, i.e., large enough to involve the true measurement, but small compared to the whole observation region, under the scale parameters being set as*β=3∼5.

## 5. Simulations

In this section, the optimal sub-pattern assignment (OSPA) miss-distance [35] is chosen as the tracking performance metric, which captures the cardinality error, as well as the distance of individual elements, of two finite sets. The OSPA distance of two finite sets $X=\left\{x_{1},\ldots ,x_{m}\right\}$ and $Y=\left\{y_{1},\ldots ,y_{n}\right\}$ is given by

(65) $${\bar{d}}_{p}^{\left(c\right)}\left(X,Y\right):=\{\begin{array}{ll} {\left(\frac{1}{n}\left(\underset{\pi \in \Pi_{n}}{\min}\sum_{i=1}^{m}d^{\left(c\right)}{\left(x_{i},y_{\pi \left(i\right)}\right)}^{p}+c^{p}\left(n-m\right)\right)\right)}^{\frac{1}{p}}, & m\leq n\\ {\bar{d}}_{p}^{\left(c\right)}\left(Y,X\right), & m>n \end{array},$$

where $\Pi_{k}$ is the set of permutations on {1,2,…,k},k>0, and the distance $d^{\left(c\right)}\left(x,y\right):=\min\left(c,\left\|x-y\right\|\right)$, while p≥1 is the order of the distance and c is the cut-off parameter.

To achieve reliable results, simulation results were acquired by averaging the outcomes of 200 Monte Carlo trials.

### 5.1. Availability of the Threshold Method

**Figure 3:**
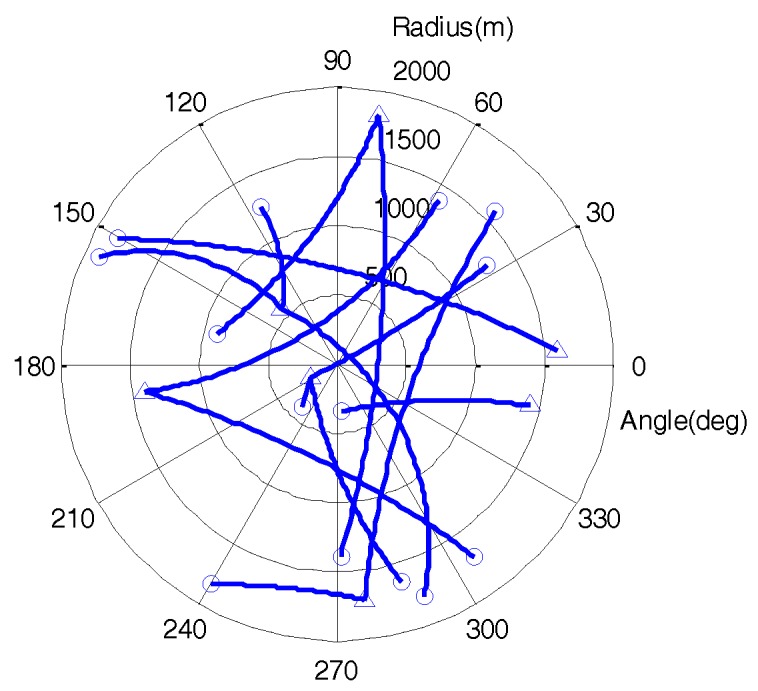
Target trajectories in polar coordinates with start and stop positions denoted by △ and ○.

This subsection describes the design an experiment to demonstrate the effectiveness of the proposed threshold method. In the simulation, the observation region was a circle with a diameter of 4000 m, while the kinematic and the measurement models were both non-linear. Fourteen targets appeared successively in the scenario, where 12 targets were newborn in an unknown position and moment. Figure 3 shows the true trajectories of all targets in the scenario.

The kinematic model employed a constant turn model, where the position and turn rate process noises followed zero-mean Gaussian distributions with standard deviations of $\sigma_{w}=20{\;m/s}^{2}$ and $\sigma_{u}=\pi /180\;\mathrm{rad}/s$, respectively. The measurement model exported range and bearing values, where the range and bearing measurement noises also followed zero-mean Gaussian distributions with standard deviations of $\sigma_{r}=20\;m$ and $\sigma_{\theta }=\pi /180\;\mathrm{rad}$, respectively. The clutter followed a uniform distribution, and the number of the clutter followed a Poisson distribution with a mean value of 10. The survival and detection probabilities were $p_{S,k}\left(x\right)=0.95$ and $p_{D,k}\left(x\right)=0.95$, respectively. Each Bernoulli component was expressed by 1000 particles.

The fast adaptive target birth intensity filter proposed in Section 4 was compared with the adaptive target birth intensity filter proposed in Section 3, by applying different thresholds with scale parameters chosen from β=1∼5. The clutter density $\tilde{\kappa }$ used in the fast filters could be determined from Equation (53) and were expressed as 0.68268κ, 0.95449κ, 0.99730κ, 0.99993κ, and 0.99999κ, respectively.

**Figure 4:**
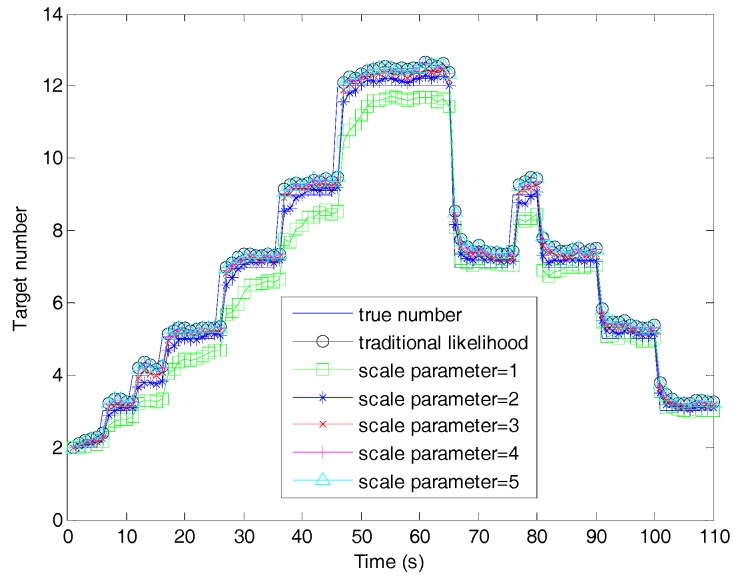
Target number estimation of the sequential Monte Carlo (SMC) adaptive target birth intensity CBMeMBer filter and the fast SMC adaptive target birth intensity CBMeMBer filter.

Figure 4 shows the estimated number of targets for the proposed filter and fast filters. It can be observed that the fast filter with the threshold of β=1 had an obvious underestimation in target number. This was because the scale parameter β=1 was too narrow to make the threshold completely contain the true measurement, which eliminated large amounts of useful likelihood calculations. The performance of the fast filter with β=2 outperformed the filters with other scale parameters, since the threshold of β=2 struck the best balance between containing the true measurement and blocking the false associated measurements. The fast filter with β=5 achieved a target number estimation most close to the filter with traditional likelihood, since the increasing threshold contained more falsely associated likelihood information. As the scale parameter increased, the fast filter asymptotically approached the filter with traditional likelihood.

**Figure 5:**
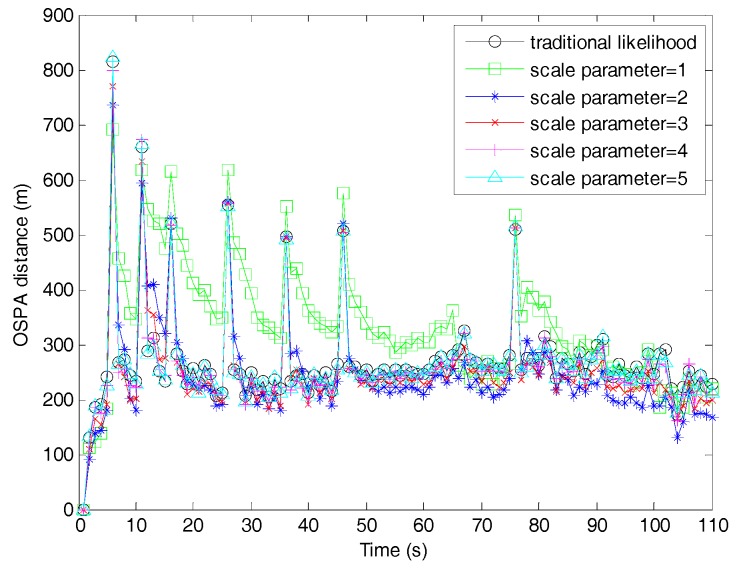
Optimal sub-pattern assignment (OSPA) distances of the SMC adaptive target birth intensity CBMeMBer filter and the fast SMC adaptive target birth intensity CBMeMBer filter.

Figure 5 compares the OSPA distances between the proposed filter and fast filters. Similar to the discussions in Figure 4, the OSPA distance of the fast filter with β=1 was the largest, and best performance could be achieved when β=2. Table 2 shows the total OSPA distance improvement of the fast filters with β=1∼5 compared to the filter with traditional likelihood. Obviously, with the scale parameter increasing, the OSPA distance of the fast filter first improved from worst to optimal, and then gradually approached that of the filter with traditional likelihood. This is consistent with the analysis made in Section 4.3 and is consistent with the trend in the target number estimation.

**Figure 6:**
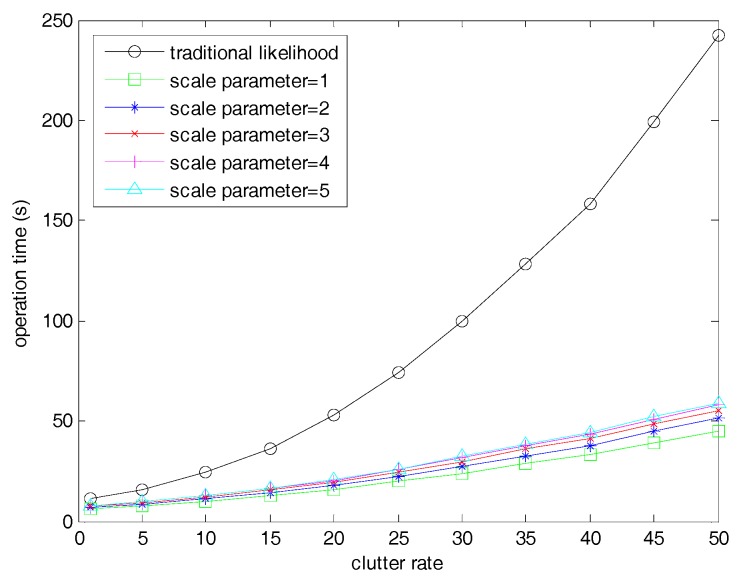
Time consumptions of the SMC adaptive target birth intensity CBMeMBer filter and the fast SMC adaptive target birth intensity CBMeMBer filter with clutter rate varied from 1 to 50.

Figure 6 shows the operation time of different filters when the clutter rate varied from 1 to 50. We can observe that the threshold method greatly reduced the time consumption of the proposed adaptive target birth intensity filter, especially in high clutter rates. When the clutter rate was 50, the time consumption of the proposed filter was reduced by about 400%. In the fast filters, the computing time increased slowly with the increase of the scale parameter but still remained at the same level. This is because all of the thresholds with given scale parameters were very small compared with the whole observation region, as shown in Equation (64). If the threshold was greatly increased to an extremely large number such as in β=100, the computing time was similar or equal to the filter with traditional likelihood.

The simulations demonstrate that the proposed fast filter can significantly reduce the operation time, which restricts the practical application of the proposed filter with traditional likelihood and can slightly improve the tracking accuracy. In the given scenario, the threshold with β=2 was the best, while other thresholds with larger scale parameters were also suitable for the filter and could help achieve similar tracking performance. Of course, too large thresholds were not included, which resulted in the fast filter having no difference with the filter adopting traditional measurement likelihood.

### 5.2. Fast SMC Adaptive Target Birth Intensity CBMeMBer Filter

In this subsection, we illustrate the advantages of the proposed fast CBMeMBer filter in adapting unknown newborn targets. Under the same scenario as in Section 5.1, the fast adaptive target birth intensity filter was compared to the filters with the known, fixed, adaptive target birth intensities and the adaptive target birth density. The scale parameter in the fast filter was chosen as *β* = 2. The modeling of different birth models is shown in Table 3, where detailed descriptions on the fixed birth intensity and the adaptive birth density can be found in References [6,13], respectively.

**Figure 7:**
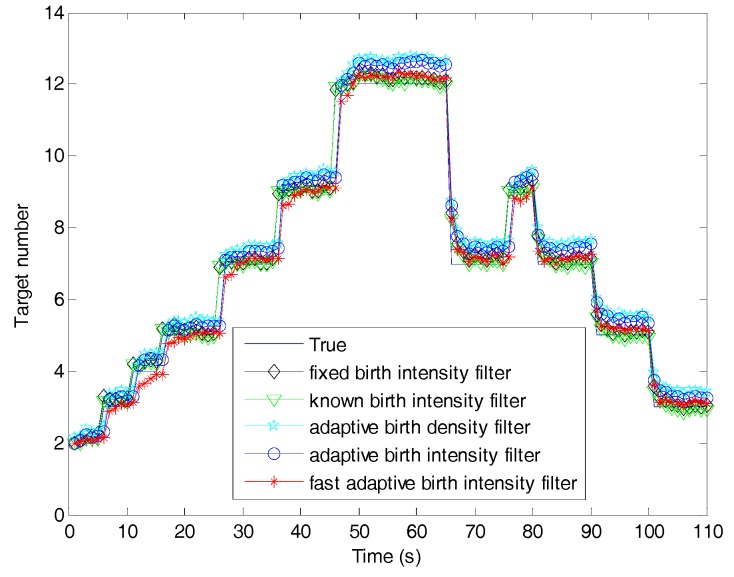
Target number estimation of the fast adaptive target birth intensity CBMeMBer filter and other CBMeMBer filters with different target birth models.

**Figure 8:**
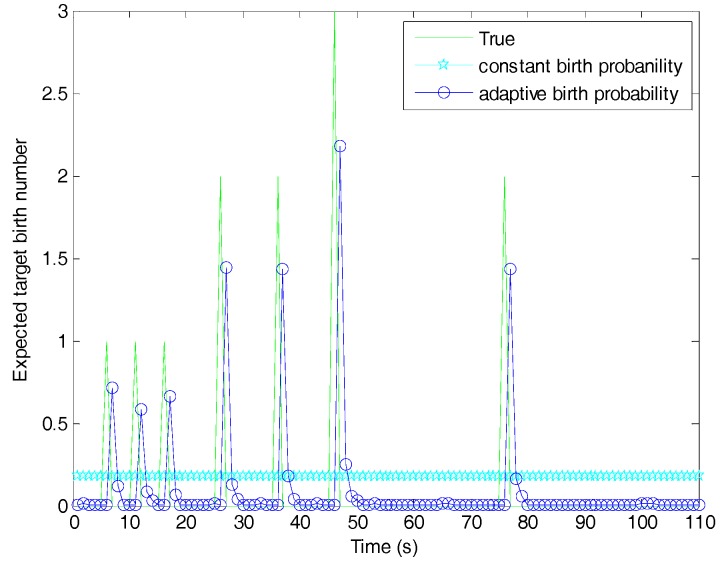
Expected number of newborn targets in constant and adaptive target birth probabilities.

Figure 7 shows the estimated number of targets versus time for the fast filter and the filters with different target birth models. The estimated number of the fixed birth intensity filter was slightly larger than the known birth intensity filter due to the fact that the known birth intensity, requiring all information about newborn targets, was optimal. The three filters using adaptive birth models had one time-step delay when capturing newborn targets, since the birth densities in these filters were generated by previous measurements. It is obvious that our proposed adaptive birth intensity filter outperformed the adaptive birth density filter. This was because, in the proposed adaptive birth intensity, a pre-processing procedure was introduced to model the birth probability, which resulted in the expected number of newborn targets being closer to the truth, as shown in Figure 8. Both of these filters had larger target number estimation than the fixed birth intensity filter, because the measurement information contained in the birth components could be regarded as a supplement to the targets in updating. The fast adaptive birth intensity filter further improved the performance of the adaptive birth intensity filter in target number estimation, due to the reasons explained in Section 5.1. Excluding time steps where newborn targets appeared, the fast adaptive birth intensity filter achieved a nearly equivalent target number estimation as the fixed birth intensity filter.

**Figure 9:**
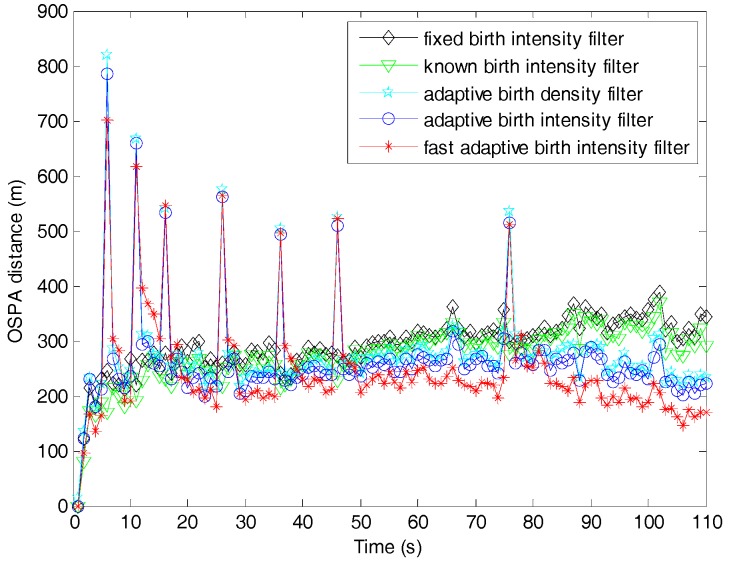
OSPA distances of the fast adaptive target birth intensity CBMeMBer filter and other CBMeMBer filters with different target birth models.

Figure 9 compares the OSPA distances of the filters under consideration. In time steps where targets appeared, the three filters with adaptive birth models were much worse than the known and fixed birth intensity filters in OSPA distance due to the delay in capturing newborn targets. Similar to Figure 7, the proposed adaptive birth intensity filters outperformed the adaptive birth density filter, and the known birth intensity filter outperformed the fixed birth intensity filter. Excluding time steps with newborn targets appearing, the filters with adaptive birth models had lower OSPA distances than the known and fixed birth intensity filters, which was opposite to the comparison of target number estimation. This was because the adaptive birth models supplied extra measurement information which led to a gain in target number estimation but a correction in target state estimation. In these three filters, the fast adaptive birth intensity filter achieved the best performance in OSPA distance.

**Figure 10:**
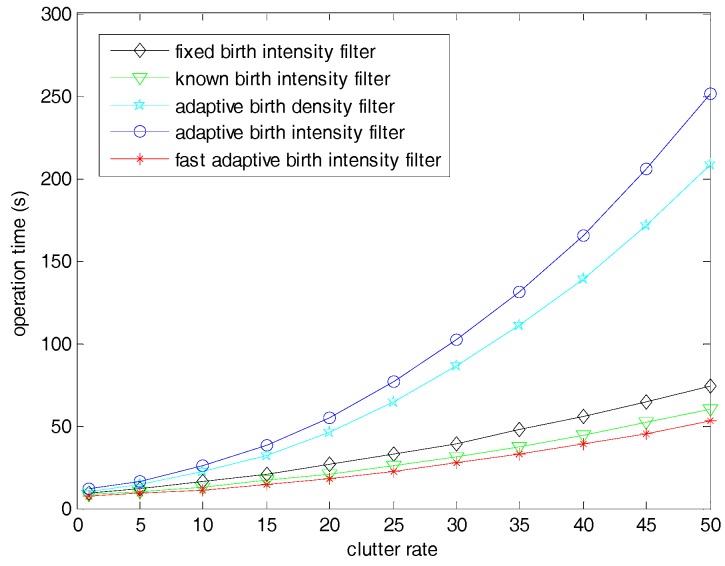
Time consumptions of the fast adaptive target birth intensity CBMeMBer filter and other CBMeMBer filters with different target birth models with clutter rates ranging from 1 to 50.

Figure 10 shows the operation time of the fast filter and the filters with different birth models. The fixed birth intensity filter ran slower than the known birth intensity filter, since it included more birth components which appeared in each filtering step. The adaptive birth intensity filter ran slower than the adaptive birth density filter, since it introduced a new pre-processing step to model the birth probability. Obviously, the two filters with the adaptive birth intensity and density had very huge time consumptions, especially under dense clutter backgrounds. This was because the adaptive birth models applying measurements to capture newborn targets were very intricate in updating. Table 4 shows the time consumptions of different filters with a clutter rate of 50. By employing the threshold, the fast adaptive birth intensity filter greatly reduced the operation time; thus, it achieved a good real-time property which was even slightly better than the known and fixed birth intensity filters.

The simulations demonstrated that the fast adaptive target birth intensity CBMeMBer filter can improve the tracking performance of newborn targets without prior birth information, and it can operate well with a good processing efficiency.

### 5.3. Fast SMC Adaptive Target Birth Intensity CBMeMBer Filter under Specific Conditions

**Figure 11:**
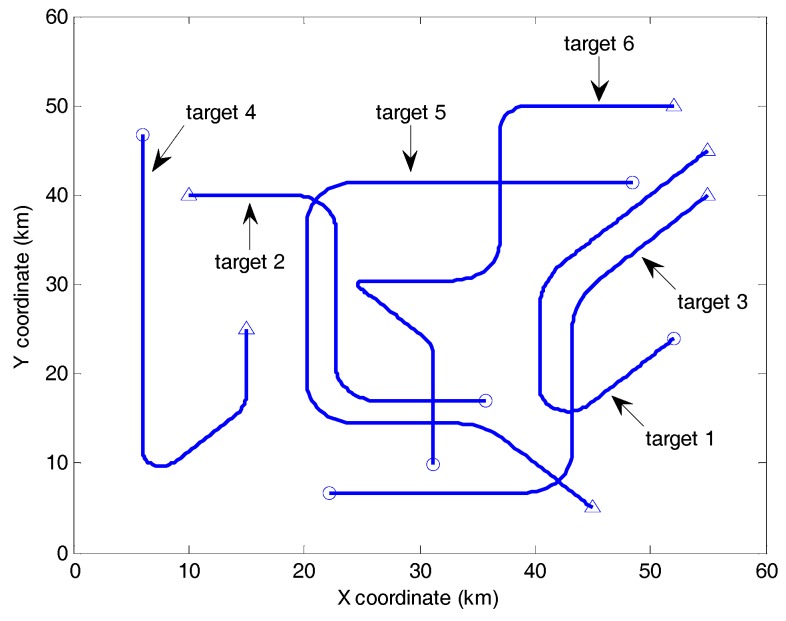
Trajectories in Cartesian coordinates with start and stop positions denoted by △ and ○.

In this subsection, simulations are presented to further demonstrate the validity and the practical application scope of the fast CBMeMBer filter. Firstly, the the fast filter and the filters with different target birth models were compared in another scenario which included intensive target maneuvering, e.g., thrusting, accelerating, and sharp turning. The high-maneuver scenario was generated by integrating the six targets of Reference [36]. Figure 11 shows the true target trajectories in this scenario.

To adapt the maneuvering of targets, multiple target motion models including constant velocity (CV), constant acceleration (CA), and constant turn (CT) models were applied. In practice, unexpected maneuvering, i.e., none of the models matching with the motion under consideration, is often encountered. To test the filters in this case, we supposed that the sharp turning, i.e., the second turn of target 4 from time 86 to 100, and the third and fourth turns of target 6 from time 116 to 125, and from time 156 to 160, was unexpected.

**Figure 12:**
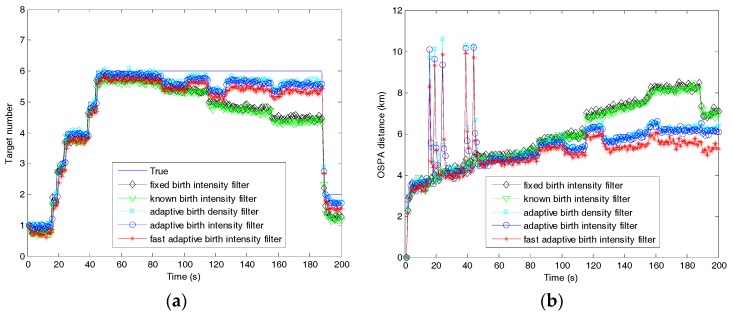
Tracking performance of the fast adaptive target birth intensity CBMeMBer filter and other CBMeMBer filters with different target birth models in the case of unexpected maneuvering: (**a**) target number estimation; (**b**) OSPA distances.

Figure 12 shows the tracking performance of the filters in the case of unexpected sharp target turning. It can be observed that the tracking performance of the filters declined due to the maneuvering of targets, especially when unexpected maneuvering happened. The obvious decline in tracking performance of the filters with the fixed and known birth intensities happened at times 86, 116, and 125 until the falsely tracked targets disappeared at time 188. Different from these two filters, the tracking performance of the filters with adaptive birth models improved after the disappearance of the unexpected maneuvering, and the fast filter outperformed its counterparts. This was because the measurement information in the adaptive birth components of the fast filter could be seen as a best initiation when the motion models were matched with the maneuvering again.

After this, another simulation was performed. The scenario adopted was the same as that in Figure 3 in Section 5.1 and Section 5.2, except that this experiment included continuous missing of sensor detections, which is always encountered in real-world tracking scenarios, such as targets being sheltered, sensor interference, active stealth, etc. In this simulation, we set a surviving target from time 36 to 110 with three short moments missing detection from time 41 to 43, and another surviving target from time 46 to 110 with 10 long moments missing detection from time 51 to 60.

**Figure 13:**
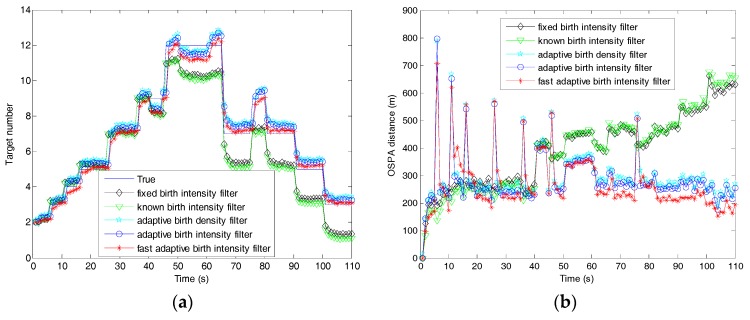
Tracking performance of the fast adaptive target birth intensity CBMeMBer filter and other CBMeMBer filters with different target birth models in the case of continuously missing detections: (**a**) target number estimation; (**b**) OSPA distances.

Figure 13 shows the tracking performance of the filters in the case that continuous missing detection occurred. We can observe that the tracking performance of the filters with the fixed and known birth intensities declined after time 40 and further declined after time 50, although the measurements were obtained again at time 44 and 61, respectively. These two filters missed the targets thoroughly once the continuous missing detection occurred. Conversely, the tracking performance of the filters with the adaptive birth models only declined at time 41 to 44 and time 51 to 61, where the decline at time 44 and 61 was the one-step delay explained in Subsection 5.2. At time 45 to 50 and after time 61, the tracking performance quickly recovered when sensor detections were obtained again, where the fast filter performed best among them.

Under these specific conditions whereby unexpected target maneuvering happened and sensor detections were continuously missed, which is common in the practical applications, the proposed fast filter with the adaptive birth intensity was valid and outperformed other compared filters. Considering the real-time ability demonstrated in Subsection 5.2, the proposed fast filter is a highly adaptable filtering method.

## 6. Conclusions

In this paper, an RFS-based fast adaptive target birth intensity Bayes filter was applied under a multi-Bernoulli framework. By modeling the target birth density using previous measurements [13] and calculating the target birth probability in a pre-processing step, the tracking performance could be improved without prior information requirements. By applying an improved threshold-based measurement likelihood, the computational burden introduced by the adaptive target birth intensity could be greatly reduced and the tracking performance could be further improved. The proposed fast adaptive target birth intensity CBMeMBer filter can be applied in most tracking applications and can be implemented as efficiently as the standard CBMeMBer filter.

## Figures and Tables

**Table 1:** Modeling of the target birth probability.

• Input: $Z_{k-1}$, ${\hat{B}}_{\Gamma ,k}$, $\pi_{k-1}$, $Z_{k}$.
• Output: $r_{\Gamma ,k-1}^{\left(i\right)},\;i=1,\ldots ,\left|Z_{k-1}\right|$.
Step 1: $p_{\Gamma ,k-1}^{\left(i\right)}\left(\cdot |z_{i}\right),\;i=1,\ldots ,\left|Z_{k-1}\right|$ according to $Z_{k-1}$ [13]
${\hat{r}}_{\Gamma ,k-1}^{\left(i\right)}={\hat{B}}_{\Gamma ,k}/\left|Z_{k-1}\right|$;
Step 2: $\pi_{P,k|k-1}$ according to $\pi_{k-1}$, Equations (6) and (7);
Step 3: ${\tilde{r}}_{L\Gamma ,k-1}^{\left(i\right)}$ according to $p_{\Gamma ,k-1}^{\left(i\right)}\left(\cdot |z_{i}\right)$, ${\hat{r}}_{\Gamma ,k-1}^{\left(i\right)}$ with Equation (20);
${\hat{r}}_{U\Gamma ,k-1}^{\left(i\right)}$ according to $p_{\Gamma ,k-1}^{\left(i\right)}\left(\cdot |z_{i}\right)$, ${\hat{r}}_{\Gamma ,k-1}^{\left(i\right)}$, $\pi_{P,k|k-1}$ with Equation (21)
${\hat{r}}_{\Gamma ,k-1}^{\left(i\right)}={\hat{r}}_{L\Gamma ,k-1}^{\left(i\right)}+{\hat{r}}_{U\Gamma ,k-1}^{\left(i\right)}$, i.e., Equation (19);
Step 4: $r_{\Gamma ,k-1}^{\left(i\right)}=\min({\hat{r}}_{\Gamma ,k-1}^{\left(i\right)},r_{\min}),\;i=1,\ldots ,\left|Z_{k-1}\right|$, i.e., Equation (25).

**Table 2:** Total optimal sub-pattern assignment (OSPA) distance improvement of different fast filters.

Fast filters	β=1	β=2	β=3	β=4	β=5
Improvement	−24.37%	8.33%	6.82%	2.73%	1.19%

**Table 3:** Modeling of known, fixed, adaptive target birth intensities and adaptive target birth density.

Target Birth Models	Birth Position	Birth Probability
known birth intensity	true	True
fixed birth intensity	six possible appearing areas	constant, $r_{\Gamma ,k}^{\left(i\right)}=0.03,\;i=1\ldots 6$
adaptive birth density	previous measurement areas	constant, $B_{\Gamma ,k}=0.18$
adaptive birth intensity	previous measurement areas	adaptively modified

**Table 4:** Operation time of different filters with a clutter rate of 50.

Filtering Methods	Fixed Birth	Known Birth	Adaptive Birth Density	Adaptive Birth	Adaptive Birth and Threshold
Opreation time	73.8975 s	59.7363 s	208.6728 s	251.4441 s	52.7258 s
